# Prevalence and burden of no-toilet households in India: an analysis of 261,746 households in 36 states/Union Territories in 2022–2023

**DOI:** 10.1080/16549716.2025.2511351

**Published:** 2025-06-19

**Authors:** Anoop Jain, Akhil Kumar, Rockli Kim, S. V. Subramanian

**Affiliations:** a Environmental Health, Boston University School of Public Health, Boston, MA, USA; b Faculty of Arts and Science, University of Toronto, Toronto, Canada; c Division of Health Policy & Management, College of Health Science, Korea University, Seoul, South Korea; d Harvard Center for Population and Development Studies, Cambridge, MA, USA; e Department of Social and Behavioral Sciences, Harvard T.H. Chan School of Public Health, Boston, MA, USA

**Keywords:** India, sanitation, no-toilet households, household consumption expenditure survey, inequality

## Abstract

India’s Swachh Bharat Abhiyan was a nation-wide program aimed at providing households with toilets to eliminate open defecation. Between 2016 and 2021, millions of households gained access to a toilet. However, as of 2021, over 238 million people still did not have a toilet, and there was considerable variation in this outcome between India’s states and Union Territories. We update the estimates on the number of no-toilet households in India using India’s Household Consumption Expenditure Survey from 2022 to 2023. We find that 12.5% of India’s households, most of which are in rural communities, still have no toilet. This amounts to over 162 million people still living without a toilet. Over 70% of those without a toilet live in just six states, and the lowest socioeconomic status households are the least likely to have a toilet. This emphasizes the need for policy makers to target states and socioeconomic groups that are lagging. Furthermore, policy makers should understand that a household’s toilet status can fluctuate because of adverse climate events, such as flooding. This evidence highlights the need for accurate and up-to-date data on no-toilet households throughout India.

## PAPER CONTEXT

- **Main findings:** As of 2023, 12.5% of India’s households do not have a toilet, amounting to over 162 million people.
- **Added knowledge:** We found that over 70% of those without a toilet live in just six states, and the lowest socioeconomic status households are the least likely to have a toilet.
- **Global health impact for policy action:** Accurate and up-to-date data on no-toilet households throughout India can help policy makers to target states and socioeconomic groups that are lagging.

## Background

The Indian government launched Swachh Bharat Abhiyan (SBA) in 2014, a nation-wide program aimed at ending open defecation throughout the country. At the time, approximately 30% of India’s population – almost 400 million people – were defecating in the open due to the lack of toilets [1]. SBA was designed to prevent open defecation by helping households gain access to toilets. SBA did this by leaning heavily on an information, education, and communication (IEC) campaign aimed at spurring demand for toilets. This IEC campaign utilized mass media campaigns and behavior change workshops to change attitudes, knowledge, and beliefs about the importance of toilet use and the dangers associated with open defecation to motivate toilet construction [2]. Additionally, SBA offered a reimbursement of INR 12,000 (138 US dollars in 2025) to households after toilet construction to further incentivize toilet adoption and use [2].

Evidence from India’s National Family Health Survey (NFHS) shows that the share of households living without a toilet fell considerably between 2016 and 2021, from 39.8% to 17.8% [3]. Nevertheless, considerable between and within-state variation in the share of households without a toilet persists throughout India, a fact shown in previously published literature [4–6]. For instance, almost 38% of households in Bihar lacked a toilet in 2021 while only 2.9% of households in Haryana did not have a toilet [3]. Additionally, there are concerns about SBA’s ability to sustain sanitation improvements throughout India over time [7–9].

The Government of India’s Household Consumption Expenditure Survey (HCES) from 2022 to 2023 provides data allowing for an updated appraisal of who still does not have a toilet in India ten years since the launch of SBA. This is an important contribution to the literature as it provides the most recent update on between and within-state variations in household toilet access thereby providing insights into where sanitation efforts need to be sustained. We provide updated estimates of the share of no-toilet households (NTH) at the all-India level, by indicators of socioeconomic status (SES), and for each state/Union Territory (UT). We also examine within-state and between-district inequality in NTH. Understanding toilet coverage gaps throughout India is crucial given the immense public health implications of inadequate access to safe sanitation in terms of both physical and mental health [10–15].

## Methods

### Study design

This study used data from the HCES in 2022–23. We used the HCES instead of data from the 2021 National Family Health Survey or from the 78th/79th rounds of the National Sample Survey due to its recency, comprehensive national coverage, and detailed socioeconomic variables. The HCES was a three-visit, multi-stage stratified panel survey covering all states and union territories (UTs) in India. First, villages and urban blocks were selected as first stage units (FSU) in each state/UT. The number of FSUs selected was proportional to the state/UT population, and FSUs were drawn from the Urban Frame Survey (UFS) or the list of villages as per the 2011 census excluding those villages included in the UFS. Then, 18 households from each FSU were selected with simple random sampling without replacement. A complete description of the sampling strategy is described in the HCES field guide [16]. We only use data collected from the household characteristic questionnaire (HCQ) that was completed on the first visit to each household. The HCES data also included a multiplier which was constructed from the complete household listing and was used in the generation of all our estimates.

### Study population

The study population consisted of all households and people living within those households surveyed on the first visit, which resulted in 261,746 households and 1,123,902 household members. All households had complete information on toilet status, and there were no missing values. Using the household multiplier included in the dataset, we estimate that this data is representative of 284,398,419 households and 1,213,886,464 people in India.

### Outcomes

The HCES survey ascertained a household’s toilet access status by asking the following question: ‘*What is the type of access of the household to latrine?*’ and each household responded with one of the following options: *exclusive use of household, common use of households in the building, public/community latrine without payment, public/community latrine with payment, no access to latrine*, and *others*. We classified households that responded with ‘*no access to latrine*’ as a ‘no-toilet household’ (NTH), and all members of that household also inherited this classification. Currently, the only analogous terminology in the World Health Organization’s Joint Monitoring Programme for Water Supply, Sanitation and Hygiene (JMP) is ‘open defecation’. However, in India, approximately 8% of those who reported defecating in the open in 2021 had access to a household toilet [3]. Thus, our term NTH refers specifically to the condition in which a household does not have access to a toilet, including community or shared toilets.

### Analysis

We first assigned each household the NTH status and then applied that status to all members living within that household. We also calculated the monthly per capita consumption expenditure for each household following the method described in the HCES factsheet [17]. Educational attainment and caste of the head of household were also extracted from the data to assess socioeconomic inequality in NTH at the all-India level. Next, we estimated the weighted prevalence of NTH by these characteristics and for each state//UT stratified by rural and urban communities. Weights were applied to our prevalence estimates so that our results are representative of each state/UT. We also estimated the headcount of NTH individuals in each state/UT by first multiplying the NTH indicator by the household size and then multiplying it by the survey weight. We then took the sum of this value stratified by each state/UT. The India headcount estimate was taken as the sum of the state/UT estimates.

Finally, we used a Markov Chain Monte Carlo (MCMC) procedure to estimate a two-level multi-level model that took the form

(1) $$\mathrm{logit}\left(Pr_{\mathrm{ikl}}\right)=\beta_{0}+\left(v_{0\mathrm{kl}}+f_{0l}\right)$$

where $\beta_{0}$represents the constant, and $v_{0\mathrm{kl}}$, and $f_{0l}$ are the residual differentials for districts *k* and states *l*, respectively. These estimates were derived using the *runmlwin* command in Stata 18 [18]. The MCMC residuals were used in the equation

(2) $$\exp[\beta_{0}+\left(v_{0\mathrm{kl}}+f_{0l}\right)]/[1\;+\exp\left(\beta_{0}+\left(v_{0\mathrm{kl}}+f_{0l}\right)\right]$$

to calculate the precision-weighted estimates for the prevalence of NTH in each district. We used this modeling approach to derive the district-level prevalence estimates because the survey is not representative at the district level. We then calculated the standard deviation of district prevalence values by each state/UT to better understand within-state and between-district NTH inequality.

## Results

The HCES contained data from 261,746 households throughout India of which 155,014 were in rural communities while 106,732 were in urban communities. Additionally, there were a total of 1,123,902 individuals sampled in the HCES. These households and individuals were sampled from each of India’s 36 states and Union Territories.

**Figure 1.**
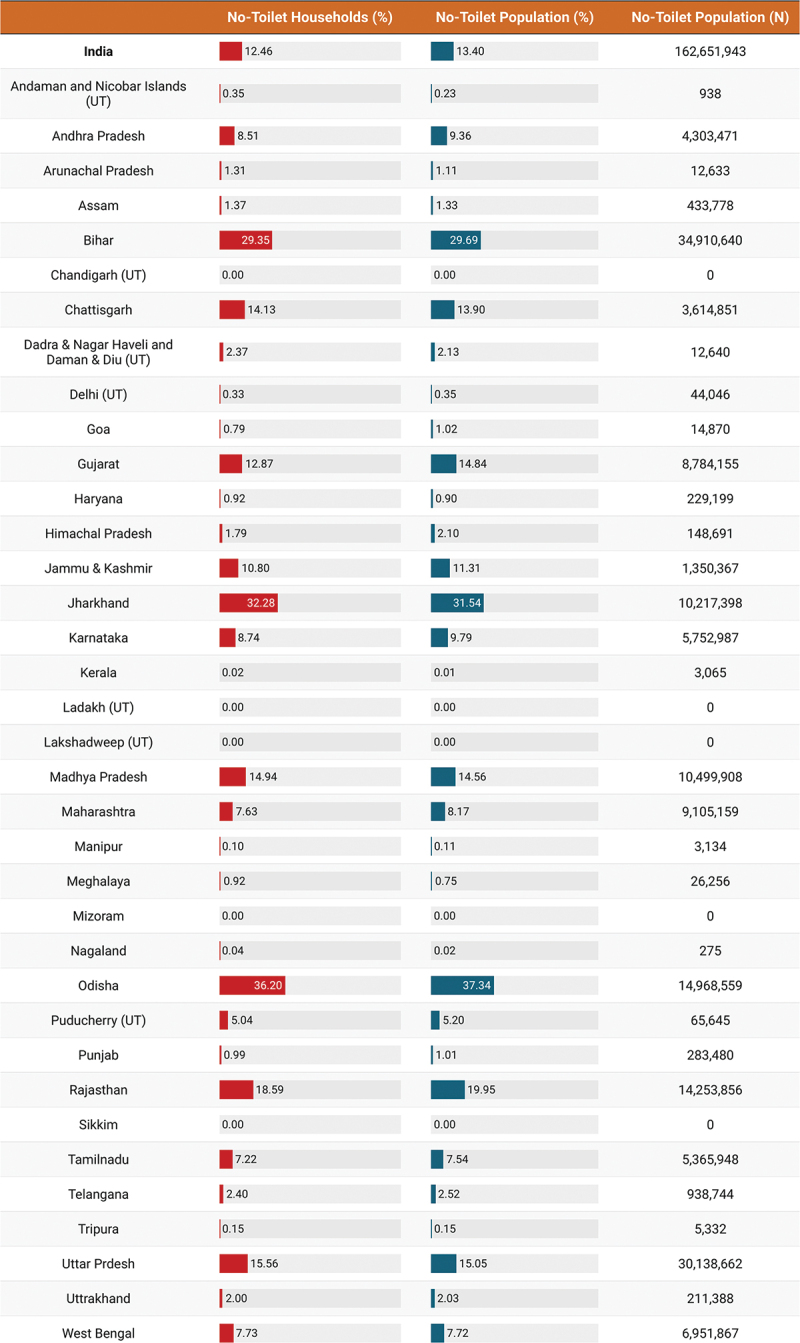
Weighted percentage NTH, and the weighted population headcount of NTH by India and state/UT.

**Figure 2.**
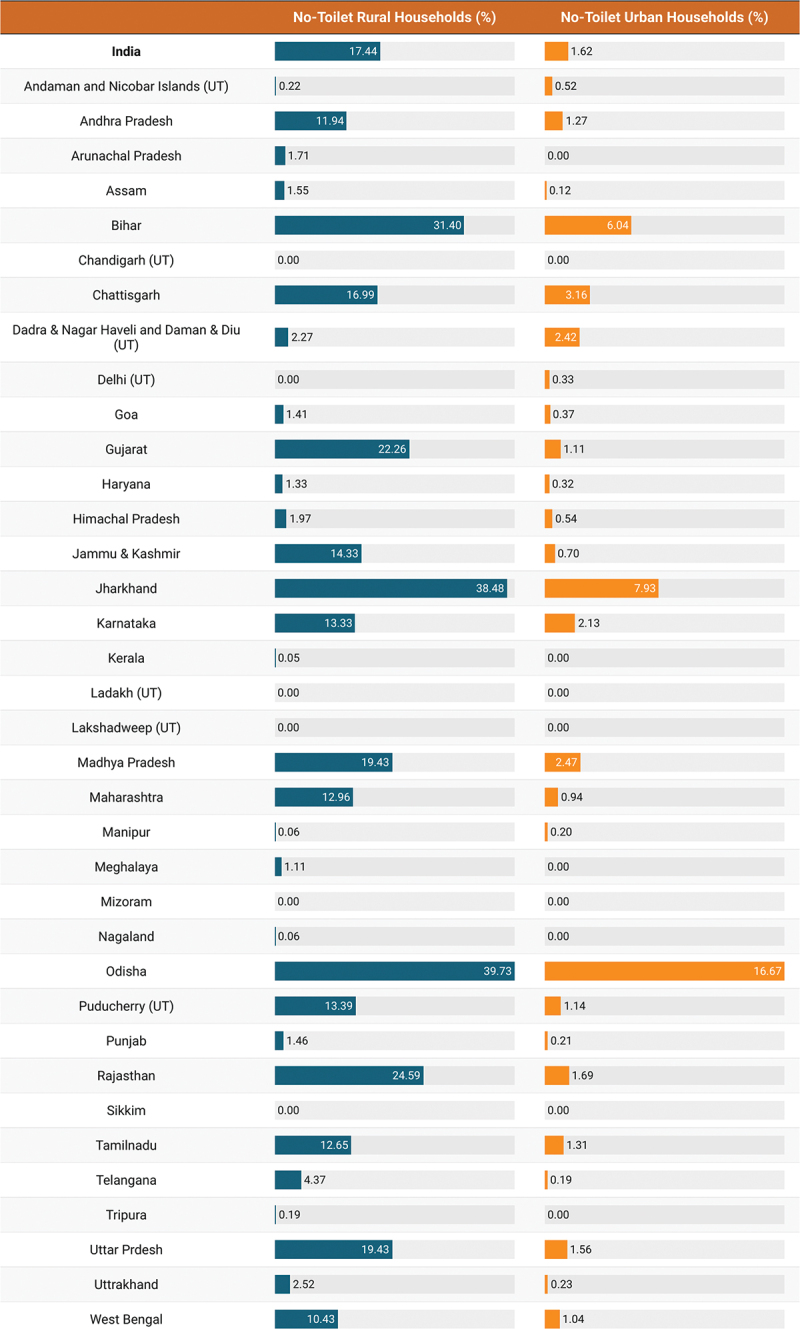
Weighted percentage of NTH by India and state/UT (overall, rural and urban).

We found 26,193 NTH households in the HCES sample and this corresponded to 120,522 individuals. The NTH prevalence was approximately 12.5% in India in 2023, while the population prevalence was 13.4% corresponding to 162,651,943 individuals (Figure 1). However, the NTH prevalence was much larger in rural communities (17.4%) than in urban communities (1.6%). In fact, approximately 93% of NTH were in rural communities (Figure 2). We also found a socioeconomic gradient in NTH. Households in the lowest monthly consumption quintile were the least likely to have a toilet (27.9%, 95% CI: 27.5% to 28.3%), as were those in which the head of household was not literate (23.3%, 95% CI: 22.9% to 23.6%) or belonged to a Scheduled Caste (19.1%, 95% CI: 18.8% to 19.5%) or Scheduled Tribe (29.1%, 95% CI: 28.6% to 29.5%). These results are presented in Table 1.

**Table 1.** Weighted prevalence of no-toilet households by markers of socioeconomic status.

	n (percent)	Percent no-toilet households 95% CI)
**Monthly per capita consumption expenditure**		
Less than 2830 INR	52,350 (20.0%)	27.9% (27.5% to 28.3%)
2830 INR to 3823 INR	52,350 (20.0%)	17.7% (17.4% to 18.0%)
3824 INR to 5080 INR	52,350 (20.0%)	10.7% (10.4% to 10.9%)
5081 INR to 7314 INR	52,350 (20.0%)	0.2% (0.1% to 0.3%)
Above 7315 INR	52,350 (20.0%)	0.1% (0.1% to 0.2%)
**Education**		
Not literate	61,596 (23.5%)	23.3% (22.9% to 23.6%)
Literate without formal education	7,940 (3.0%)	15.6% (14.8% to 16.4%)
Below primary	18,327 (7.0%)	13.9% (13.5% to 14.5%)
Formal education up to higher secondary	137,356 (52.5%)	8.9% (8.7% to 9.0%)
Graduation and above	36,527 (14.0%)	1.8% (1.6% to 1.9%)
**Caste**		
Scheduled Tribe	38,084 (14.6%)	29.1% (28.6% to 29.5%)
Scheduled Caste	44,549 (17.0%)	19.1% (18.8% to 19.5%)
Other Backwards Caste	104,461 (39.9%)	11.0% (10.8% to 11.2%)
Other	73,416 (28.1%)	3.9% (3.8% to 4.1%)
Not reported	1,236 (0.4%)	4.7% (3.5% to 5.8%)
**Religion**		
Hindu	201,769 (77.1%)	13.7% (13.5% to 13.8%)
Muslim	32,016 (12.2%)	6.9% (6.7% to 7.3%)
Christian	17,696 (6.8%)	7.8% (7.4% to 8.2%)
Sikh	4,023 (1.5%)	0.6% (0.4% to 0.9%)
Jain	548 (0.2%)	0.8% (0.06% to 1.5%)
Buddhist	3,434 (1.3%)	9.8% (8.8% to 10.9%)
Zoroastrian	37 (0.01%)	0%
Other	2,146 (0.8%)	16.8% (15.2% to 18.4%)
Not reported	77 (0.03%)	0%

Note: The values in the first column are the unweighted sample characteristics. The values in the second column represent the weighted prevalence of NTH by each SES category. The cutoff ranges for the monthly per capita consumption expenditure represent the first to fifth quintiles of these values in the sample. The education variable listed in this table is the highest education attained by the head of the household. Caste is also the caste of the head of the household. We note that Scheduled Castes, Scheduled Tribes, and Other Backwards Castes are categories that include India’s historically marginalized social groups.

**Figure 3.**
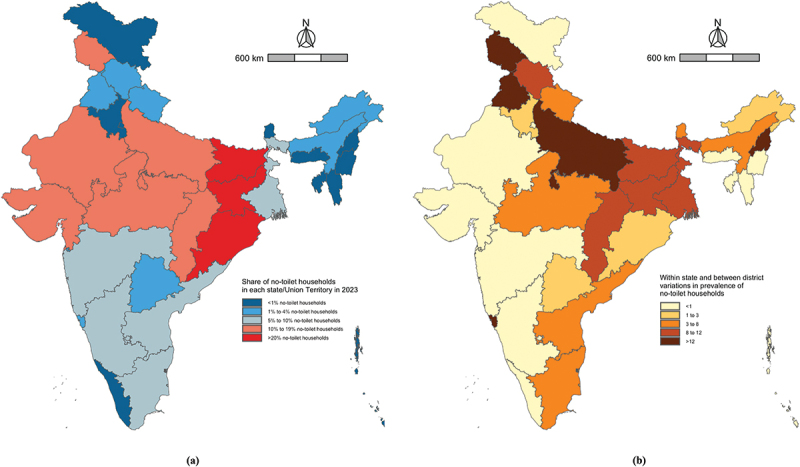
A. Map depicting the weighted mean percentage of NTH by state/UT in 2023. B. Map depicting between-district and within-state variations in the prevalence of NTH.

Note: The values presented in panel (B) are standard deviations of the district-level prevalence values of NTH for each state/Union Territory.

The prevalence of NTH varied considerably between states and Union Territories both in terms of household and population prevalence. The prevalence of NTH was above 30% in both Odisha and Jharkhand but below 1% in 15 states and Union Territories, including Punjab, Haryana, Delhi, and Kerala. The population prevalence of NTH was highest in Odisha at 37.3%. But, as of 2023, of the almost 163 million people without a toilet in India, approximately 71% live in just six states. These are Bihar, Uttar Pradesh, Odisha, Rajasthan, Madhya Pradesh, and Jharkhand. Over ten million people do not have toilet in each of these states. In Bihar alone, almost 35 million people did not have a toilet as of 2023. The population prevalence of NTH was zero in five states and Union Territories as of 2023. These were Chandigarh, Sikkim, Mizoram, Lakshadweep, and Ladakh. We found that as of 2023, the urban NTH prevalence was above 5% in just three states (Odisha, Jharkhand, and Bihar). These results are presented in Figure 3.

Finally, we found within-state and between-district variation in NTH as measured by the standard deviation of district prevalence values within states/UTs. The standard deviation of these values was greatest in Jammu & Kashmir, Uttar Pradesh, Punjab, Goa, and Nagaland (Figure 3). We also found considerable between-district variation in Bihar, Chhattisgarh, Himachal Pradesh, Jharkhand, Lakshadweep, and West Bengal (Figure 3).

## Discussion

Using India’s HCES from 2023, we show that NTH remain throughout India. Approximately 12.5% of households reported not having a toilet which corresponds to approximately 162,651,943 individuals. The burden of NTH remains the highest in rural communities and those in the lowest socioeconomic groups. Additionally, over 70% of those without a toilet live in just six states which are Bihar, Uttar Pradesh, Odisha, Rajasthan, Madhya Pradesh, and Jharkhand. We also found that the prevalence of NTH was above 10% in Gujarat, Chhattisgarh, and Jammu & Kashmir indicating the policy makers in these areas need to continue prioritizing programs aimed at improving household toilet coverage. Finally, we show between-district variations in the household prevalence of NTH in certain states and UTs, an indication of within-state inequality.

There are two data-related limitations associated with this study. First, the HCES was not designed to be representative at the district level. Hence, the district-level estimates were produced using our MCMC models, which we then used to assess within-state variations in NTH. Second, our study does not consider toilet quality. This is important because some households might rely on open pits/drains or hanging latrines. These low-quality toilets provide little public health protection. However, we only considered the total absence of a household toilet in this study.

These results are important for several reasons. First, we show that as of 2023, over 162 million people in India did not have a toilet. This is lower than what was reported using different data in 2021 [3]. Second, our results show that rural areas continue lagging behind the most, a finding consistent with previous results [3], and one that shows that India’s rural residents remain physically and socially isolated from decision makers [19]. We also found that those in India’s lowest socioeconomic groups also remain at highest risk of living in households without a toilet, a result consistent with findings from previous work [3,20]. Third, our results highlight the importance of up-to-date subnational reporting of NTH. The state of Rajasthan declared itself to be open defecation free in 2018. However, we show that as of 2023, almost 19% of Rajasthan’s residents did not have a toilet in 2023, corresponding to over 14 million people. Updated reporting helps ensure that policy makers continue targeting vulnerable populations.

Updated reporting is also important because a household’s toilet status can change over time. Findings from other studies show that toilets built under Swachh Bharat Abhiyan can become dysfunctional or low-quality, which deters use [7,21,22]. Adverse climate events can also impact toilet status. Flooding can cause irreparable damage to household toilets making a household’s toilet ownership status tenuous. Regular and up-to-date reporting should account for these fluctuations in household toilet ownership over time and by region that might result from climate-related events [23].

In conclusion, our results show that over 162 million people in India lived in households without a toilet in 2023. However, work remains to be done. We show that efforts should target rural areas along with certain priority states and districts where over 100 million people still do not have a toilet and where the prevalence of NTH is above 10%. Efforts need to also continue prioritizing India’s most socioeconomically marginalized communities that are still the least likely to have a household toilet. Furthermore, up-to-date and accurate reporting that relies on independent third-party data is critical so that progress is not incorrectly overestimated and that vulnerable populations are not left behind. Up-to-date reporting is also important given that adverse climate events can cause irreparable damage to household toilets thereby changing the status of whether a family does or does not have access to safe sanitation.

## Data sharing statement

The datasets generated during and/or analyzed during the current study are available from the corresponding author on reasonable request. The data can be requested from this link: https://microdata.gov.in/nada43/index.php/catalog/226.
